# 1441. Hand hygiene compliance in Brazil: from rich hospitals in the southwest to jungle hospitals - the big challenge

**DOI:** 10.1093/ofid/ofad500.1278

**Published:** 2023-11-27

**Authors:** Braulio Couto, Estevão Urbano Silva, Guenael Freire Souza, Shirley Ferreira, Erika Vrandecic, Louranny Góis, Maria Luiza Peixoto, Thais Santos, Virginia Andrade, Carolina Vieira, Jeruza Romaniello, Luciana Covello, Thaís Couto, Angela Vieira, Anna Elisa Fonseca, Thiago Novato, Vanessa Silva, Amanda Maia, Jéssica Macêdo, Matheus Pereira, Thalita Lima, Camila Zampa, Fabrizia de Paula, Jacqueline Soares, Janete Ferreira, Sara Carmos, Rafael Fernandes, Hoberdan Pereira, Eder Hideki Munefiça, Lorraine Mantovanelli, Gabrielle Mota, Kehone M Miranda, Carlos E Starling

**Affiliations:** Biobyte Tecnologia em Epidemiologia, Belo Horizonte, Minas Gerais, Brazil; Hospital Madre Teresa, Belo Horizonte, Minas Gerais, Brazil; Fundação Hospitalar Nossa Senhora de Lourdes, Belo Horizonte, Minas Gerais, Brazil; Fundação Hospitalar Nossa Senhora de Lourdes, Belo Horizonte, Minas Gerais, Brazil; Biocor Instituto, Nova Lima, Minas Gerais, Brazil; Biocor Instituto, Nova Lima, Minas Gerais, Brazil; Biocor Instituto, Nova Lima, Minas Gerais, Brazil; Biocor Instituto, Nova Lima, Minas Gerais, Brazil; Hospital Madre Teresa, Belo Horizonte, Minas Gerais, Brazil; Hospital Evangélico, Belo Horizonte, Minas Gerais, Brazil; Hospital Evangélico, Belo Horizonte, Minas Gerais, Brazil; Hospital Evangélico, Belo Horizonte, Minas Gerais, Brazil; Hospital Evangélico, Belo Horizonte, Minas Gerais, Brazil; Hospital Felício Rocho, Belo Horizonte, Minas Gerais, Brazil; Hospital Felício Rocho, Belo Horizonte, Minas Gerais, Brazil; Hospital Felício Rocho, Belo Horizonte, Minas Gerais, Brazil; Hospital Felício Rocho, Belo Horizonte, Minas Gerais, Brazil; Hospital Madre Teresa, Belo Horizonte, Minas Gerais, Brazil; Hospital Madre Teresa, Belo Horizonte, Minas Gerais, Brazil; Hospital Madre Teresa, Belo Horizonte, Minas Gerais, Brazil; Hospital Madre Teresa, Belo Horizonte, Minas Gerais, Brazil; Hospital Metropolitano Odilon Behrens, Belo Horizonte, Minas Gerais, Brazil; Hospital Metropolitano Odilon Behrens, Belo Horizonte, Minas Gerais, Brazil; Hospital Metropolitano Odilon Behrens, Belo Horizonte, Minas Gerais, Brazil; Hospital Metropolitano Odilon Behrens, Belo Horizonte, Minas Gerais, Brazil; Hospital Metropolitano Odilon Behrens, Belo Horizonte, Minas Gerais, Brazil; Hospital Municipal Odilon Behrens, Belo Horizonte, Minas Gerais, Brazil; Hospital Municipal Odilon Behrens, Belo Horizonte, Minas Gerais, Brazil; Hospital Regional Adamastor Teixeira Oliveira, Vilhena, Rondonia, Brazil; Hospital Regional Adamastor Teixeira Oliveira, Vilhena, Rondonia, Brazil; Hospital Universitário Ciências Médicas (HUCM), Belo Horizonte, Minas Gerais, Brazil; Hospital Universitário Ciências Médicas (HUCM), Belo Horizonte, Minas Gerais, Brazil; Sociedade Mineira de Infectologia - SMI, Belo Horizonte, Minas Gerais, Brazil

## Abstract

**Background:**

Our study seeks to address three important questions: (a) What is the current rate of HH adherence in Brazilian hospitals? (b) Which of the five moments of HH has the lowest adherence rate in Brazilian hospitals? (c) What is the HH compliance rate, as measured by direct observation, among physicians, nurses, nursing technicians, and other healthcare professionals? And (d) is there a difference in HH adherence rates when comparing private hospitals to public/philanthropic hospitals?

**Methods:**

We conducted covert, random daily observations to assess hand hygiene compliance in 8 Brazilian hospitals over three months (Jan-Mar/2023). Observations were conducted in both critical and non-critical care units, and data on compliance rates were collected in the SACIH 3i system (https://nsp.sacihweb.com). To calculate the compliance rate, we divided the number of observations in which hand hygiene was performed correctly when necessary by the total number of observed instances where hand hygiene was required.

**Results:**

We conducted observations on 4,662 hand hygiene opportunities across 8 hospitals in three months. The global hand hygiene adherence rate varied between hospitals, ranging from 14% to 74% (Fig. 1), with a median adherence rate of 57% for Brazilian hospitals. The first moment, before touching a patient, had the lowest adherence rate of 47% (Fig. 2). Nursing technicians were the most frequently observed professional group (69%), with compliance rates of physicians = 51%, nurses = 65%, nursing technicians = 62%, and other healthcare professionals = 54% (p-value < 0.001). There was no difference in the overall hand hygiene rate between private and public/philanthropic hospitals, both with a compliance rate of 60%. However, significant differences were found between hospital types in Moment 1, Moment 2, and Moment 5 (Tab.1 ).

**Figure 1:**
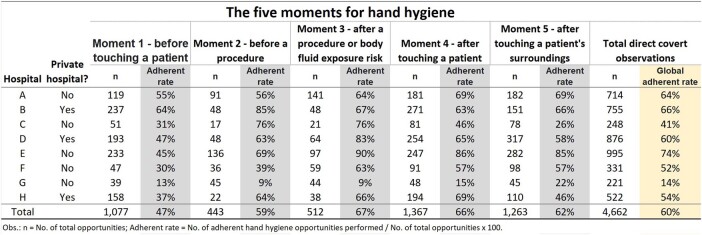
Hand hygiene adherence in Brazilian hospitals (2023): comparing global rates and adherence across the five moments.

**Figure 2:**
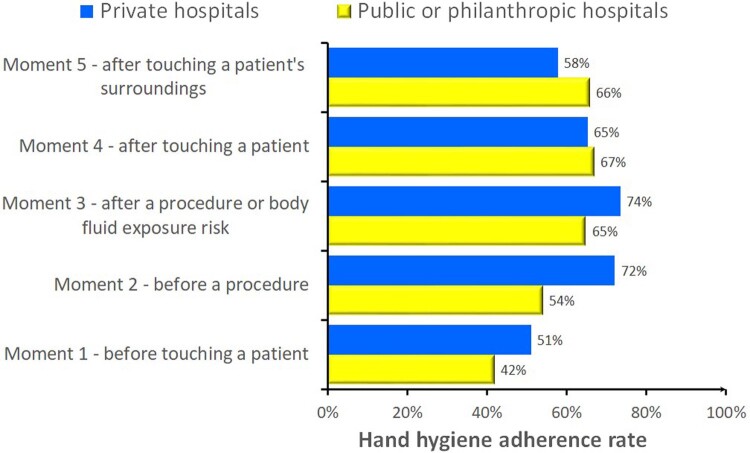
Hand hygiene adherence rates in Brazilian hospitals (2023): private vs. public/philanthropic hospitals by the five moments.

**Table 1:**
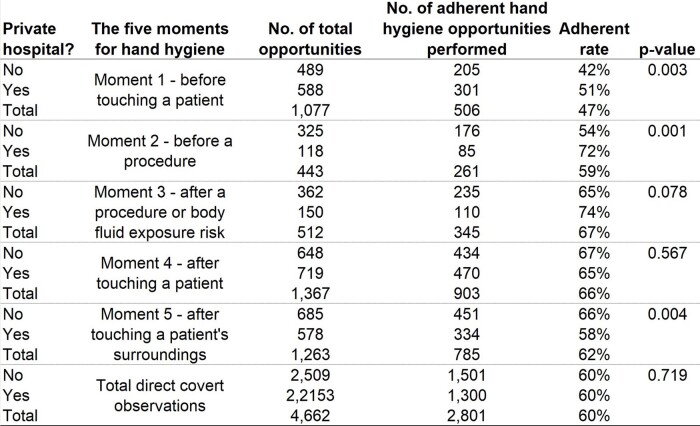
Hand hygiene adherence rates in Brazilian hospitals (2023): private vs. public/philanthropic hospitals compared across five moments.

**Conclusion:**

Hand hygiene adherence in Brazilian hospitals remains low at 47%. The first moment, before touching the patient, has the lowest adherence rate, indicating that professionals prioritize their own safety over patients. Physicians have the lowest adherence rate, and private hospitals have better adherence than public/philanthropic hospitals.

**Disclosures:**

**All Authors**: No reported disclosures

